# Investigation of Gamma-Ray Shielding Properties of Bismuth Oxide Nanoparticles with a Bentonite–Gypsum Matrix

**DOI:** 10.3390/ma16052056

**Published:** 2023-03-02

**Authors:** Mahmoud I. Abbas, Ahmed M. El-Khatib, Mohamed Elsafi, Sarah N. El-Shimy, Mirvat F. Dib, Hala M. Abdellatif, Raqwana Baharoon, Mona M. Gouda

**Affiliations:** 1 Physics Department, Faculty of Science, Alexandria University, Alexandria 21511, Egypt; 2 Clinical Oncology and Nuclear Medicine Department, Faculty of Medicine, Alexandria University, Alexandria 21511, Egypt; 3 Nuclear Medicine at Mediclinic, Airport Road Hospital, Abu Dhabi 48481, United Arab Emirates

**Keywords:** bentonite, shielding, SEM, LAC, mass attenuation coefficient, nano-bismuth oxide, Z_eff_, EABF, EBF

## Abstract

Due to the present industrial world, the risk of radioactivity is notably increasing. Thus, an appropriate shielding material needs to be designed to protect humans and the environment against radiation. In view of this, the present study aims to design new composites of the main matrix of bentonite–gypsum with a low-cost, abundant, and natural matrix. This main matrix was intercalated in various amounts with micro- and nanosized particles of bismuth oxide (Bi_2_O_3_) as the filler. Energy dispersive X-ray analysis (EDX) recognized the chemical composition of the prepared specimen. The morphology of the bentonite–gypsum specimen was tested using scanning electron microscopy (SEM). The SEM images showed the uniformity and porosity of a cross-section of samples. The NaI (Tl) scintillation detector was used with four radioactive sources (^241^Am, ^137^Cs, ^133^Ba, and ^60^Co) of various photon energies. Genie 2000 software was used to determine the area under the peak of the energy spectrum observed in the presence and absence of each specimen. Then, the linear and mass attenuation coefficients were obtained. After comparing the experimental results of the mass attenuation coefficient with the theoretical values from XCOM software, it was found that the experimental results were valid. The radiation shielding parameters were computed, including the mass attenuation coefficients (MAC), half-value layer (HVL), tenth-value layer (TVL), and mean free path (MFP), which are dependent on the linear attenuation coefficient. In addition, the effective atomic number and buildup factors were calculated. The results of all of these parameters provided the same conclusion, which confirms the improvement of the properties of γ-ray shielding materials using a mixture of bentonite and gypsum as the main matrix, which is much better than using bentonite alone. Moreover, bentonite mixed with gypsum is a more economical means of production. Therefore, the investigated bentonite–gypsum materials have potential uses in applications such as gamma-ray shielding materials.

## 1. Introduction

Gamma radiation that emits through space as electromagnetic waves can interact with different materials to different degrees. It has many useful and beneficial applications in medicine and industry, yet it affects human life by causing cell mutations and potentially damaging organs. In addition, it affects the environment. Therefore, it is very important to protect humans and the environment from the harmful effects of this radiation. The protection depends on three main factors: time (by reducing the exposure time to radiation)**,** distance (by increasing the separation distance between the body and the radioactive source and reducing the radiation effect), and shielding, which is the preferred protection against radiation [1,2]. The material options for shielding from applications, such as accelerators, nuclear reactors, medical applications, research labs, agriculture, and industry are expanding daily. The selection of shielding materials depends on the requirement for an exposure rate reduction, source type, medium constraints, and cost-effective analysis. Moreover, the higher the atomic number and density of the shielding material/mixture, the greater the attenuation of the gamma radiation.

The traditional method of shielding against photons is lead shielding. Lead has useful properties such as being soft and malleable. Furthermore, lead is resistant to corrosion, thereby offering long-term protection, has a high density, and has high radiation shielding ability. However, lead-based shields are toxic and harmful to both humans and the environment. Another method is lead composite shielding, which is a mixture of lead with other metals that causes high attenuation for radiation and reaches a certain protection level. Composite shielding materials for radiation can be nearly equivalent to the protection levels of lead. The other technique is the lead-free shielding method, in which composite materials of metals or metal oxides increase the efficiency of the shielding material to minimize the hazards of lead materials [3,4]. For example, Ozdogan et al. [5] examined the photon attenuation efficiency and buildup factors of several borate glasses doped with Cd, Fe, V, and Bi. Çakıroğlu et al. [6] studied the radiation attenuation coefficients of different proportions of additives (Fly ash, silica fume, and polypropylene fiber) produced in dry mixture shotcrete, both by experimental processes and by the deep neural network based on DBN. Further, Akman et al. [7] investigated the gamma-ray photon interaction characteristics for a variety of materials, such as calcium silicide, magnesium silicide, magnesium boride, calcium hexaboride, aluminum oxide, and titanium dioxide. Recently, many studies have been concerned with using nanoparticle materials to improve the shielding capabilities of certain materials against radiation.

A post-transition metal with one of the lowest thermal conductivity values among all metals is bismuth (Z = 83). The density of the free element is 86% that of lead. The most significant bismuth compound in the industry is Bi_2_O_3_. The use of micro- and nanosized Bi_2_O_3_ powders as environmentally benign radiation shielding materials in glasses, concrete, alloys, and polymers has recently been the subject of extensive research. Tiamduangtawan et al. [8] investigated the gamma-shielding, mechanical, and self-healing properties of polyvinyl alcohol (PVA) hydrogels with the addition of varying contents of bismuth oxide (Bi_2_O_3_) (0 wt%, 20 wt%, and 40 wt%) and particle sizes (nanoparticles and microparticles) based on samples prepared using a one-cycle freezing–thawing method. Intom et al. [9] prepared natural rubber by incorporating bismuth oxide (Bi_2_O_3_) with different loading ratios. Moreover, Kurtuluş et al. [10] used waste pharmaceutical glass (PG), in an attempt to gain a value-added product for radiation shielding applications, a new glass system was fabricated with the nominal composition of x Bi_2_O_3_ – (100 − x)PG (x: 0, 5, 15, 25, and 35 wt%). However, it should be noted that point defects in bismuth oxide are practically not studied, although it is assumed that their properties should be close to such oxides as Al_2_O_3_ and Ga_2_O_3_, in which new vacancies are formed only under particle exposure (neutron, ion, and protons), whereas under X-ray or gamma rays, only electronic processes occur [11,12].

In this study, bentonite is used as a low-cost shielding material. To increase its hardness and density, bentonite was mixed with gypsum [13]. Furthermore, to increase the attenuation effect of the matrix, the bentonite–gypsum matrix is fixed with different concentrations of micro- and nanoparticles sizes of bismuth oxide (Bi_2_O_3_). The purpose of this paper was to investigate the effects of differently sized particles of bismuth oxide filler in building shielding materials and to determine the attenuation coefficient for each composite, containing various percentages of the bismuth oxide, based on particle sizes (micro and nano), ultimately, to obtain a novel shielding material that protects against gamma radiation.

## 2. Materials and Method

### 2.1. Materials

In this study, the basic material was bentonite, which was collected from Suez, Egypt. Here, it was crushed into smaller pieces and then ground into powder form. The second basic material was gypsum. The chemical compositions of bentonite and gypsum were analyzed using an Energy Dispersive X-ray (EDX), as shown in Table 1. Additionally, two different kinds of Bi_2_O_3_ particles were used: Bi_2_O_3_ nanoparticles, which were chemically prepared by the Nanotech company in Egypt, and Bi_2_O_3_ microparticles, which are a high-purity powder with 99.9% purity, and were received from Loba Chemie, India.

### 2.2. Samples Preparation

The bentonite powder, the fine gypsum powder, the filler metal oxide (Bi_2_O_3_), and water were used as mixing materials to prepare the samples. The samples were prepared by adding specific mass ratios of about (0%, 6%, 13%, and 20%) micro- and nano-bismuth oxide (Bi_2_O_3_)_._ Table 2 shows the sample codes and the corresponding weight fraction as a percentage (wt%) of bentonite, gypsum, and Bi_2_O_3_. Then, the mixture was poured into a coin-shaped mold, with a 3 cm diameter and a 0.5 cm height. Finally, each sample was left to air-dry and become cohesive in its mold. Archimedes’ method was employed to ascertain the average density (g/cm^3^) of the samples, using water as the immersion medium.

### 2.3. Morphology Test

**Figure 1:**
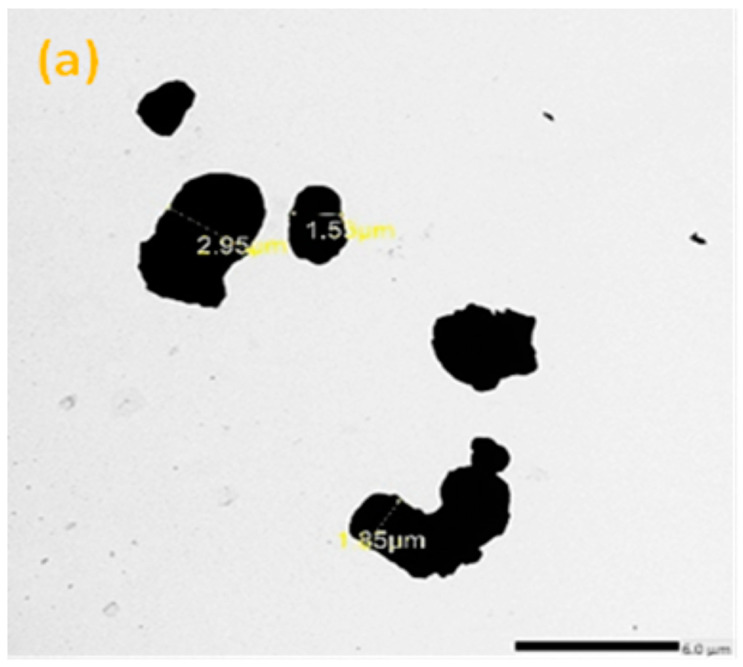

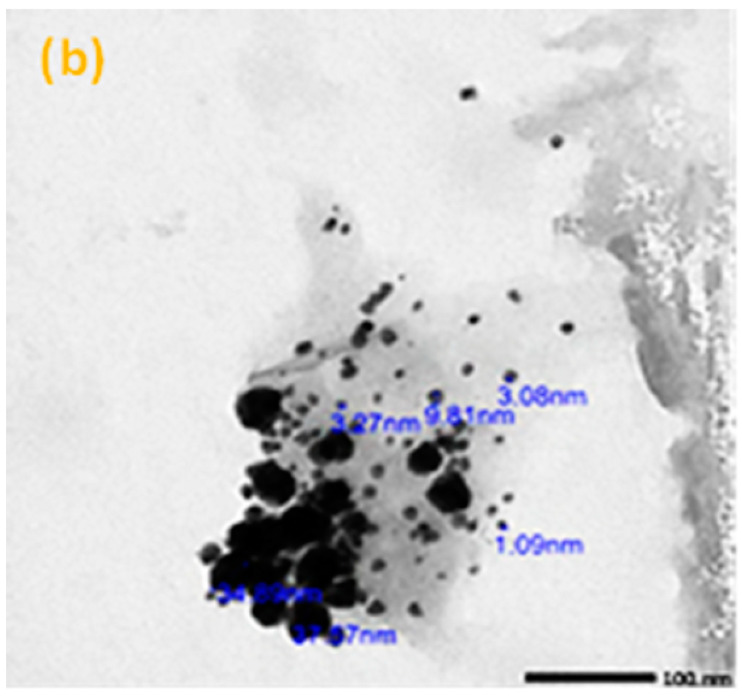
The TEM image of (**a**) micro-Bi_2_O_3_ particles, (**b**) nano-Bi_2_O_3_ particles.

**Figure 2:**
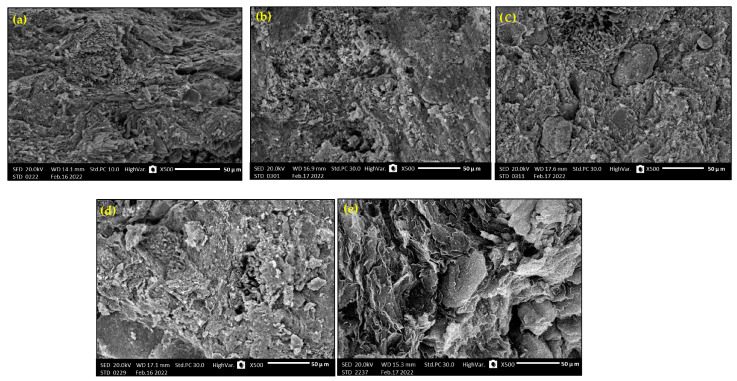
SEM images of (**a**) BG-0, (**b**) BG-1Micro, (**c**) BG-5Micro, (**d**) BG-1Nano, (**e**) BG-5 Nano.

TEM analysis was carried out using a JEM-2100F transmission electron microscope with a 200 kV acceleration voltage to identify the particle sizes of the micro- and nano-sized Bi_2_O_3_. According to Figure 1a,b, the average size of the microparticles was 3 µm, while the nanoparticles were 12 nm. Scanning electron microscopy (SEM) (JEOL-JFC-1100E) can be used to determine the cross-section morphology and distribution of the Bi_2_O_3_ inside the samples. Figure 2 illustrates some images of the scanned samples using SEM for bentonite–gypsum, 6 wt% bulk of Bi_2_O_3_/bentonite–gypsum, 20 wt% bulk of Bi_2_O_3_/bentonite–gypsum, 6 wt% nano of Bi_2_O_3_/bentonite–gypsum, and 20 wt% nano of Bi_2_O_3_/bentonite–gypsum samples. As seen in Figure 2a, the SEM image of the blank sample was smooth and clear compared to the filled composites exhibited in Figure 2b–e. In Figure 2d,e, the nano-Bi_2_O_3_ particles have a more homogeneous distribution within the main matrix (bentonite–gypsum) than the micro-Bi_2_O_3_ particles, Consequently, the nano combination performed well in terms of protection.

### 2.4. Radiation Measurements

**Figure 3:**
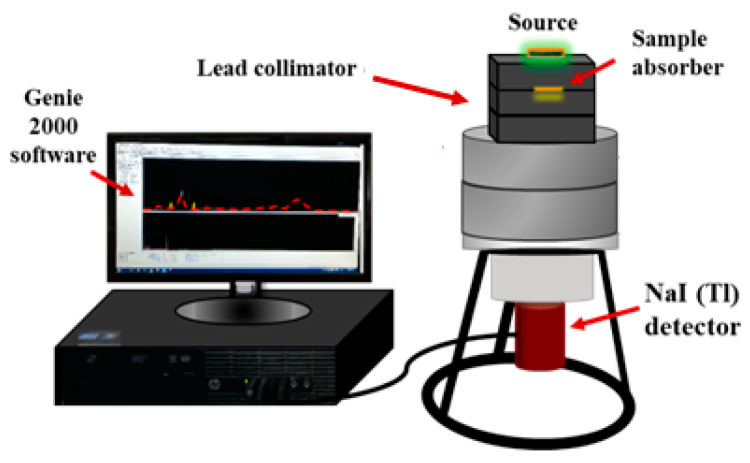
The experimental setup configuration of the sample with respect to the radioactive source and the detector.

An experiment was carried out to determine the intensity of γ-rays that penetrated the sample and the shielding parameters. The gamma source provided a narrow beam that transmitted through the sample and reached the detector. The detector used in this experiment is a scintillation detector of the type NaI (Tl). Figure 3 shows the setup configuration of the source–detector system. Four radioactive sources were used in the present work: ^241^Am, ^137^Cs, ^133^Ba, and ^60^Co. These sources emit radiation of a wide range of energies, from 0.0595 MeV to 1.332 MeV. The photons that emerged from the sample interacted with the detector crystal, which converted them into signals and displayed them as peaks in a spectrum, via the Genie 2000 software [14,15,16,17,18].

To evaluate the capability of the material for shielding, the linear attenuation coefficients (LAC) were calculated by Beer-Lambert’s law [19]:

(1) $$\mu =\frac{1}{t}\ln\left(\frac{I_{0}}{I}\right)$$

where μ is the linear attenuation coefficient (LAC), I_o_ is the intensity of the incident gamma rays emitted from the radioactive source, I is the intensity of gamma rays after being attenuated in the presence of the absorber, and t is the sample thickness.

The half-value layer (HVL) is defined as the absorption thickness needed to decrease the incident radiation on the substance to 50% of its initial value when preparing a suitable substance for protection against radiation and is calculated using Equation (2) [20,21].

(2) $$\mathrm{HVL}=\frac{\ln2}{\mu }$$

The tenth-value layer (TVL) is represented by the absorption thickness needed to decrease the incident radiation on the substance to 10% of its initial value [22,23].

(3) $$\mathrm{TVL}=\frac{\ln10}{\mu }$$

The mean free path (MFP) is defined as the average distance at which the photon travels through the sample without any interaction.

(4) $$\mathrm{MFP}=\frac{1}{\mu }$$

The mass attenuation coefficient (μ_m_) is a parameter that can be used widely in studying and comparing the efficiency of shielding different materials. It is used to measure the average number of radiation interactions with matter in a given mass thickness of the target material and is calculated by dividing the LAC of the sample by its density (⍴), as shown in the following equation [24,25].

(5) $$\mu_{m}=\frac{\mu }{\rho }$$

The relative deviations for the measured mass attenuation coefficient compared to the XCOM result (∆_1_) and between the micro- and nano-measured results (∆_2_) are given by the following equations:

(6) $$\Delta_{1}\%=\frac{{\mathrm{MAC}}_{\mathrm{XCOM}}-{\;\mathrm{MAC}}_{\mathrm{Micro}}\;}{{\mathrm{MAC}}_{\mathrm{Micro}}}\times 100$$

(7) $$\Delta_{2}\%=\frac{{\mathrm{MAC}}_{\mathrm{Nano}}\;–{\;\mathrm{MAC}}_{\mathrm{Micro}}\;}{{\mathrm{MAC}}_{\mathrm{Micro}}}\times 100$$

The effective atomic number is computed by the following equation [26].

(8) $$Z{\mathrm{eff}}_{\;}=\frac{\Sigma_{I\;}I{\;I}_{i}\;{\left[\frac{\mu }{\rho }\right]}_{i}}{Iw_{i\;}I{\left[\frac{\mu }{\rho }\right]}_{i}}$$

where Z_i_, A_i_, and w_i_ represent the atomic number, atomic weight, and the weight fraction of element ‘i’ in the composite, respectively.

The buildup factor is a correction factor (multiplicative factor) that concerns the scattered photons and the influences of the secondary particles in the medium during shielding calculations. As a result, the contributions of the scattered photons were included in this multiplicative factor. The calculation of buildup factors depends on the rate at which the photons flow through a medium and the number of interactions that occur. To calculate the energy absorption buildup (EABF) and exposure buildup (EBF) factors, the computation of the equivalent atomic number (Z_eq_) and the G–P fitting method are used for each tested composite [27,28]. Three steps should be undertaken for each composite, as follows:

(A) The computation of equivalent atomic number (Zeq):

The equivalent atomic number is energy dependent. It can be calculated by finding the ratio of Compton mass attenuation (µ/⍴), Compton, and the total mass attenuation coefficient (µ/⍴) for a given composite in the photon energy range from 0.015 to 15 MeV, using the WinXCom program [29]. The following equation is used to obtain Z_eq_:

(9) $$z_{\mathrm{eq}}=\frac{z_{1}(\log R_{2}-\log R)+z_{2}(\log R-\log R_{1\;})}{\left(\log R_{2}-\log R_{1}\right)}$$

where Z_1_ and Z_2_ are the atomic numbers that correspond to R_1_ and R_2_, respectively. R is the ratio of the given composite at a particular energy.

(B) The calculation of the G–P fitting parameters:

Then, the G–P filling parameters (a, b, c, d, and X_k_) will be obtained using the following interpolation equation.

(10) $$b=\frac{b_{1}(\log z_{2}-\log z_{\mathrm{eq}})+b_{2}(\log z_{\mathrm{eq}}-\log z_{1})}{\left(\log z_{2}-\log z_{1}\right)}$$

(C) The calculation of the buildup factor:

Finally, the buildup factors have been estimated using the following equations.

(11) $$B\;(E,\;x)=1+\frac{b-1}{K-1}\left(K^{x}-1\right),\;\mathrm{for}\;K\;\neq \;1$$

B (E, x) = 1+ (b − 1) x, for K = 1(12)

(13) $$K\left(E,x\right)={\mathrm{cx}}^{a}+d\frac{\tanh\left(\frac{x}{X_{k}}-2\right)-\tan h\left(-2\right)}{1-\tan h\left(-2\right)},\;\mathrm{for}\;x\;\leq 40\;\mathrm{mfp}$$

where K (E, x) is the variation corresponding to the change in energy, and E is the incident energy at the x mean free path.

## 3. Results and Discussion

The mass and linear attenuation coefficient values for the samples were determined for the photon energy range between 0.05953 MeV and 1.332 MeV. Table 3 shows the measured values and theoretical values using the XCOM of mass attenuation coefficients, linear attenuation coefficients, relative deviation, and density of pure bentonite–gypsum, micro-Bi_2_O_3_/bentonite–gypsum, and nano-Bi_2_O_3_/bentonite–gypsum composites.

Table 3 clearly shows that the mass attenuation coefficient (μ_m_) decreases with increasing photon energy and increases with increasing bismuth oxide Bi_2_O_3_ in the sample. The mass attenuation coefficients (μ_m_) of the samples are notable for having a large value at a photon energy of 0.05953 MeV and then decreasing gradually as the photon energy increased. This behavior can be related to the photon partial interaction process. At low photon energies (e.g., about 0.05953 MeV), the attenuation values follow the photoelectric absorption, which is inversely proportional to E^3^. At intermediatory energy, Compton scattering dominates the attenuation process, and the attenuation is inversely proportional to E (for example, at energy 59.53 keV, the photon cross-sections for photoelectric absorption and Compton scattering for BG-5Micro are 1.008 and 0.143 cm^2^/g, respectively, while at an energy of 661.66 keV, they are 0.008 and 0.074 cm^2^/g, respectively. Mass attenuation values are nearly constant for the energies equal to or higher than 1.022 MeV because the pair production process is dominant in this region. It is obvious that the attenuation ability of the sample relates to the composition of the sample, such that the gamma photons cause higher attenuation as the bismuth oxide (Bi_2_O_3_) amount in the sample increases. Further, according to Table 3, as the Bi_2_O_3_ content in the samples increases, the mass density increases, reaching 2.815 ± 0.03 g cm^−3^ in the BG-5Micro sample. The larger molecular weight and the higher photon–electron interactions of Bi_2_O_3_, increase the linear attenuation coefficient value with Bi_2_O_3_ for each energy under investigation.

The influence of Bi_2_O_3_ in micro- and nanoscales on the mass and linear attenuation coefficients for different samples were also studied. The higher MAC and LAC values for Bi_2_O_3_ NPs than for the bulk Bi_2_O_3_ are due to the distribution of particles in the sample. The smaller size of the NPs allows for a more uniform distribution of particles within the sample, thus, increasing the surface–mass ratio, and resulting in a higher probability of interaction between the Bi_2_O_3_ NPs and the gamma photons. As a result, the attenuation capabilities of the Bi_2_O_3_ NP sample are better than those of the Bi_2_O_3_ bulk sample. Moreover, Table 3 shows that the relative deviation (∆_1_%) between the theoretical values (XCOM) and the experimental micro values range between −3.72 and 2.74%, which confirms the precision of the results. The relative deviation (∆_2_%) between the experimental nano and micro values ranges between 4.7 and 16.9%, confirming that the nanoparticles improve the attenuation efficiency.

**Figure 4:**
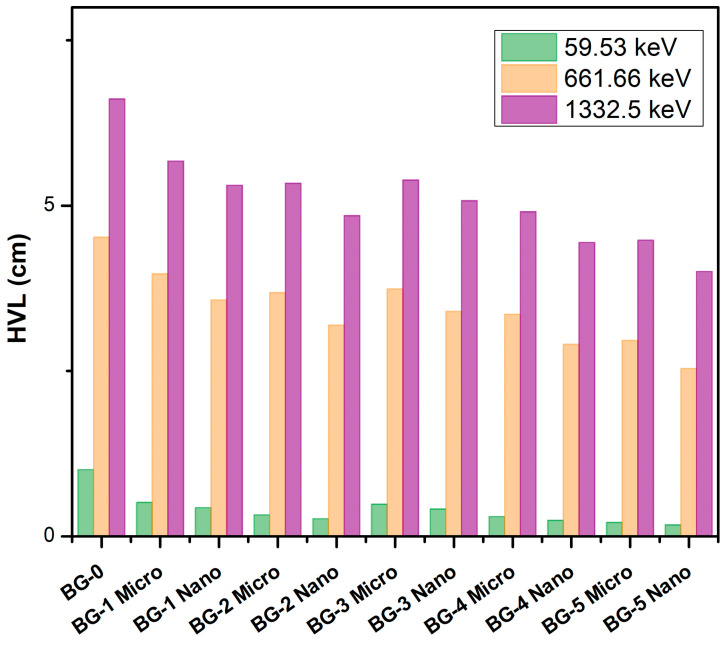
The half-value layer (HVL) of the Bi_2_O_3_/bentonite–gypsum composites at photon energies 59.53, 661.66, and 1332.5 keV.

The half-value layer values provide certain information about the shielding capability of the sample against the gamma photons (the lower the HVL, the higher the shielding efficiency). Figure 4 shows the HVL for the compositions at photon energies of 59.53, 661.66, and 1332.5 keV. The HVL for the BG-0 sample tends to increase regularly in the examined energy range. For instance, the HVL regularly rises from 1.01 cm at 59.53 keV to 4.52, and 6.61 cm at 661.66, and 1332.5 keV, respectively. The same trend is observed for the remaining samples. Furthermore, it was realized that the HVL values of BG-5, which contains 20 wt% of the bulk and NPs Bi_2_O_3_, have much lower values than the HVL of the BG-0, BG-1, BG-2, BG-3, and BG-4 samples at the same photon energies. Therefore, the greater the amount of bismuth oxide (Bi_2_O_3_) content plays an important factor in reducing the half-value layer of the investigated samples. Moreover, it was noted that the HVL levels for the nano-Bi_2_O_3_/bentonite–gypsum composites are much lower than those of micro-Bi_2_O_3_/bentonite–gypsum composites, which have the same weight percentage of bentonite, gypsum, and Bi_2_O_3_ at the same photon energies. For example, at a photon energy of 59.53 keV, the HVL of the BG-1Nano (0.44 cm) < BG-1Micro (0.54 cm), BG-2Nano (0.27 cm) < BG-2Micro (0.33 cm), BG-3Nano (0.42 cm) < BG-3Micro (0.49 cm), BG-4Nano (0.24 cm) < BG-4Micro (0.3 cm), and BG-5Nano (0.18 cm) < BG-5Micro (0.21 cm). Thereby indicating the higher performance of the nanocomposites at shielding against radiation. Furthermore, when the samples with the same Bi_2_O_3_ concentrations but different gypsum concentrations are compared, it is notable that the higher concentrations of gypsum equate to lower HVL values at the same photon energies because gypsum has a higher density than bentonite.

**Figure 5:**
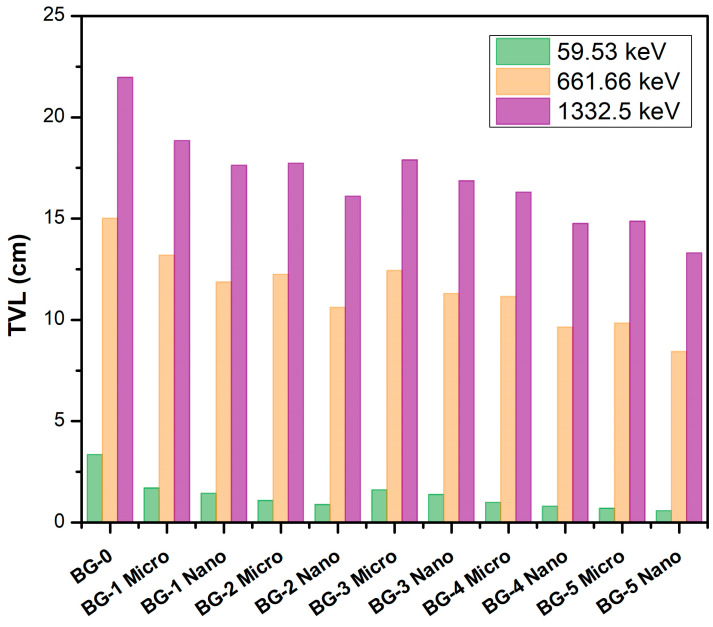
The tenth-value layer (TVL) of the Bi_2_O_3_/bentonite–gypsum composites at photon energies of 59.53, 661.66, and 1332.5 keV.

Figure 5 shows the TVL, which is energy dependent, for the composites of Bi_2_O_3_/bentonite–gypsum. It is obvious that as the photon energy increases, so do the TVL values, and more shielding material thickness is required to reduce the intensity of the incident γ-ray to one-tenth of its initial value. For example, the HVL of the BG-0 sample increases from 3.36 cm, at 59.53 keV, to 15.02 cm, at 661.66 keV, and 21.97 cm at 1332.5 keV. Moreover, this figure shows the greatest tenth-value layer for BG-0, which is a pure bentonite–gypsum sample without any Bi_2_O_3_. By adding different amounts of Bi_2_O_3_ (6 wt%, 13 wt%, and 20 wt%) to the bentonite–gypsum matrix, the values of the tenth-value layer decrease. At 59.53 keV, for example, the TVL values are equal to 3.36 cm, 1.71 cm, 1.09 cm, and 0.70 cm for BG-0, BG-1Micro, BG-2Micro, and BG-5Micro, respectively. Furthermore, the particle size influences the tenth-value layer values, with nano-Bi_2_O_3_/bentonite–gypsum samples having lower TVL values at the same photon energies as the micro-Bi_2_O_3_/bentonite–gypsum samples. When the samples with the same concentrations of Bi_2_O_3_, yet with a higher concentration of gypsum were compared, it was discovered that the ones with the higher gypsum concentration presented lower TVL values.

**Figure 6:**
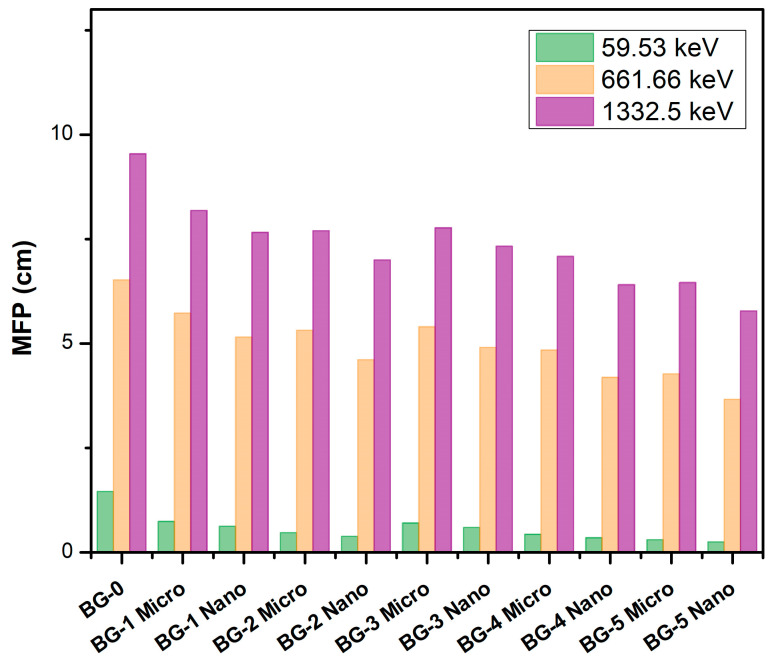
The mean free path of the Bi_2_O_3_/bentonite–gypsum composites at photon energies 59.53, 661.66, and 1332.5 keV.

The MFPs of the Bi_2_O_3_/bentonite–gypsum composites for photon energies of 59.53, 661.66, and 1332.5 keV are shown in Figure 6. The mean free path is small when the photon energy is low, and it increases as the photon energy increases. The MFP for the BG-0 sample increased from 1.46 cm to 9.54 cm when the photon energy varied from 59.53 keV to 1332.5 keV, and the MFP for the BG-5Micro sample increased from 0.31 cm to 6.46 for the same respective energies. Therefore, in the application, it is recommended to increase the sample thickness because the high photon energy can penetrate deeper in the sample. Additionally, the increment of the Bi_2_O_3_ content in the sample was shown to improve the shielding efficiency of the sample. For example, at 59.53 keV, the MFP values are equal to 1.46 cm, 0.74 cm, 0.47 cm, and 0.31 cm for the BG-0, BG-1Micro, BG-2Micro, and BG-5Micro samples, respectively. This is because at low photon energies, the photoelectric absorption interaction depends on the atomic number to the power four (Z^4^), while at high photon energies, the Compton scattering interaction relates to the atomic number (Z). The photon interaction probability increases as the atomic number (Z) increases. This means that the attenuation of photons will increase, leading to a decrease in the MFP of the used sample [30].

Another important parameter that can be used to explain the different characteristics of the material is the effective atomic number (Z_eff_). Table 4 lists the calculated Z_eff_ values (using Equation (8)) for the bentonite–gypsum specimens fixed with Bi_2_O_3_ at γ-ray energies in the range of 59.53–1332.50 keV. The Z_eff_ of the composite depends on the relative portion of the Z values of the constituent elements of each sample. By comparing the values of BG-1, which contains a smaller amount of Bi_2_O_3_, with BG-5, which contains the largest amount of Bi_2_O_3_, it was observed that the BG-5 Z_eff_ possessed higher values than that of BG-1, at the same γ-ray energies. Moreover, samples with the same Bi_2_O_3_ concentration and a higher amount of gypsum showed higher Z_eff_ values than those with smaller gypsum amounts. Where Z_eff_ of BG-1 < BG-3 and BG-2 < BG-4 at the same γ-ray energies.

**Figure 7:**
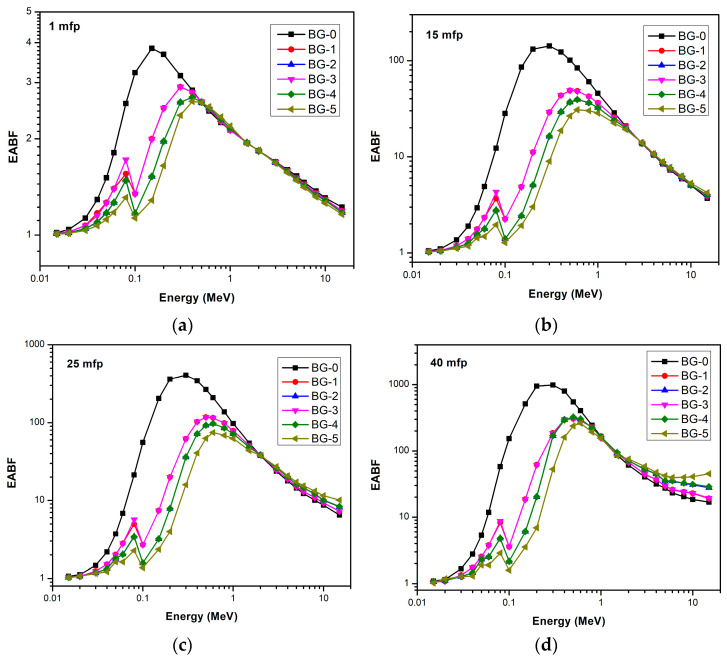
EABF as a function of photon energy at (**a**) 1mfp, (**b**)15 mfp, (**c**) 25 mfp, and (**d**) 40 mfp for bismuth oxide bentonite–gypsum.

**Figure 8:**
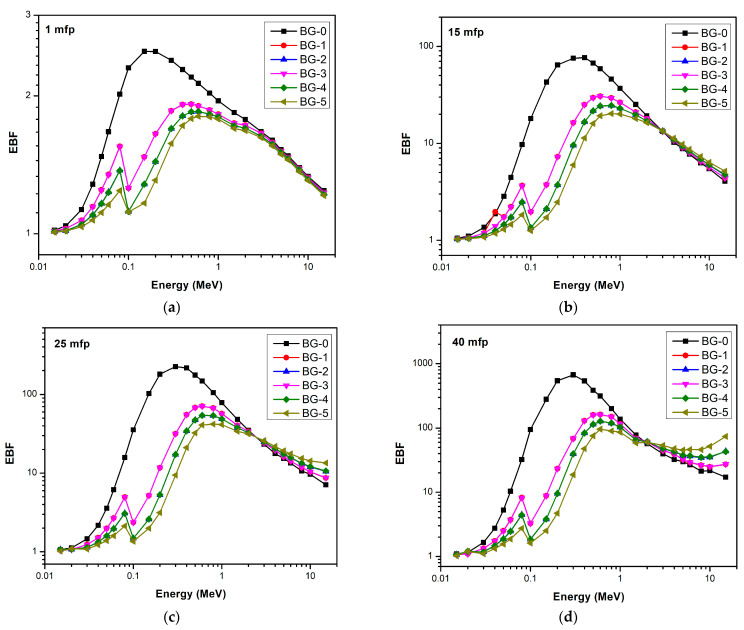
EBF as a function of photon energy at (**a**) 1mfp, (**b**)15 mfp, (**c**) 25 mfp, and (**d**) 40 mfp for bismuth oxide bentonite–gypsum.

The equivalent atomic number (Z_eq_) for bentonite–gypsum with Bi_2_O_3_ as a filler was calculated between 0.015 and 15 MeV of photon energy. After that, the EABF and EBF were calculated for all composites using the G–P fitting parameters. Figure 7 and Figure 8 represent the variation of EABF and EBF for bismuth oxide bentonite–gypsum, with photon energies for different penetration depths. It is clear that the lowest buildup factor values occur for composites that contain a higher amount of Bi_2_O_3_ at a constant mfp. Moreover, it is obvious that for all composites, as the mfp values increase, the buildup factor values rise, showing maximum buildup factor values at 40 mfp.

The variation in buildup factors with energy is because in lower photon energy regions, photoelectric absorption dominates, and in higher photon energy regions, pair production dominates. In the intermediate photon energy range, where Compton scattering dominates, the buildup factor increases. The multiple Compton scattering events increase the buildup factors to reach the maximum values. At the pair production region, the higher the penetration depth, the lower the buildup factor, where it reaches a minimum value. The sudden increase in the behavior of the buildup factor at lower energies is due to the K-edge of the bismuth oxide. The K-edge is a phenomenon that describes a sudden increase in the attenuation coefficient of photons when the energy of the incident photons becomes slightly more than the binding energy of the K-shell electron of the atoms interacting with those photons.

**Figure 9:**
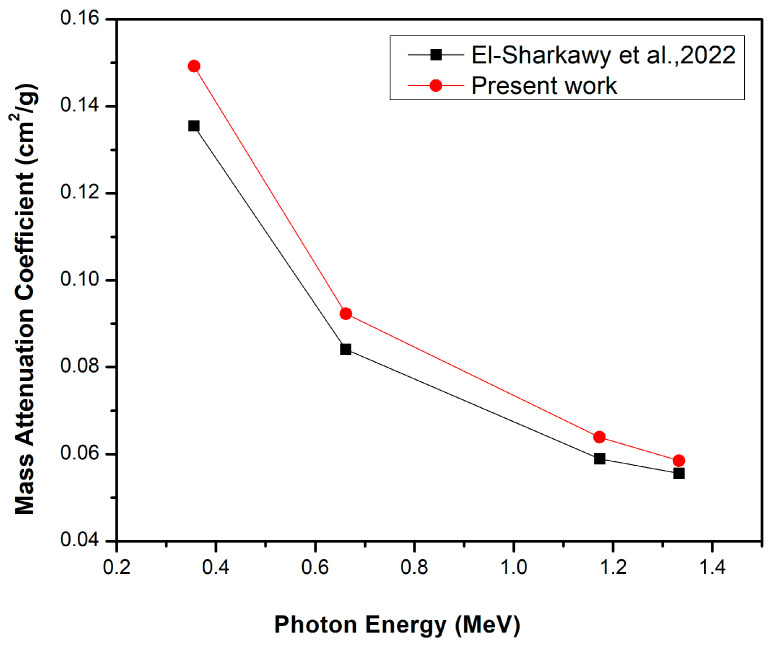
The comparison between the mass attenuation coefficient of 20 wt% nano-Bi_2_O_3_ in bentonite–gypsum matrix and the 20 wt% nano-Bi_2_O_3_ in the bentonite matrix only (Ref. [31]).

**Figure 10:**
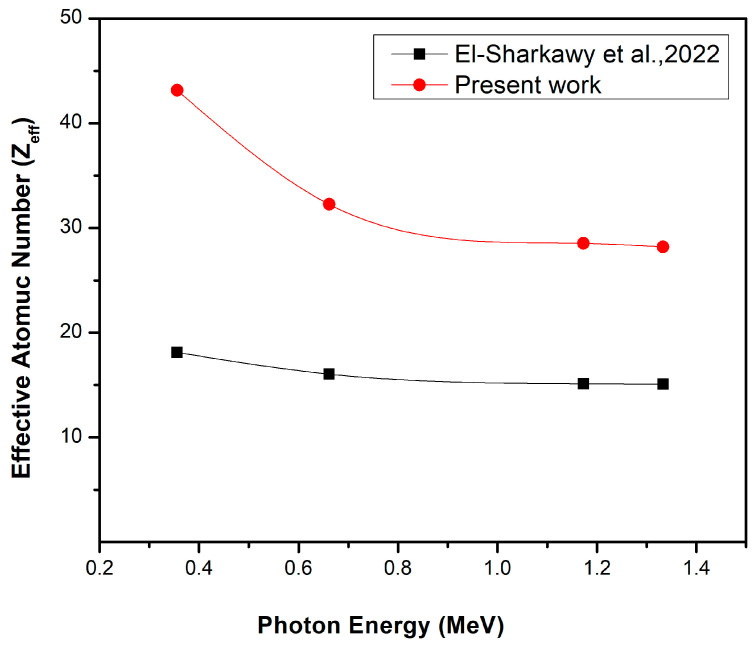
The effective atomic number of the present work (red line) and the effective atomic number of El-Sharkawy et al. (back line) [31], for the same 20 wt% Bi_2_O_3_ NPs.

Comparison of this study, which uses a bentonite–gypsum matrix and Bi_2_O_3_ NPs (filler), with El-Sharkawy et al. [31], where nanoscale Bi_2_O_3_ was used as filler in a bentonite matrix, at the same Bi_2_O_3_ NP concentrations and photon energies, highlighted that some elements in the bentonite of the present work were not found in the specimens used byEl-Sharkawy et al., such as magnesium, potassium, titanium, and iron, in addition to the chemical composition of gypsum. Consequently, the bentonite–gypsum composites used in the current work for the 20 wt% Bi_2_O_3_ NPs have a higher mass attenuation coefficient (red line) than the bentonite matrix composite used in El-Sharkawy et al. [31] (black line); see Figure 9. This means that the presence of these elements and gypsum in the nanocomposites increases the mass attenuation coefficient, which confirms the higher shielding performance of bentonite in the nano-Bi_2_O_3_ composites. Moreover, it was observed that the effective atomic number of the present work (red line) is larger than that produced by El-Sharkawy et al. [31] (black line) for the 20 wt% NP- Bi_2_O_3_, see Figure 10 for the same reason.

## 4. Conclusions

Bentonite and gypsum are used in this study as the main matrix because of their characteristics. Bismuth oxide (Bi_2_O_3_) is used in this paper as a filler with various weight percentages (6, 13, and 20) in bulk and nanosized particles to produce new bentonite-based bulk and nanocomposite radiation shielding materials. The experimental values of the mass attenuation coefficient were determined and compared with the theoretical XCOM ones, giving good comparability of the results. It was observed that the specimens with a higher weight percentage of Bi_2_O_3_ showed higher mass attenuation coefficients. Moreover, the specimens that contain nanoscale Bi_2_O_3_ have much higher mass attenuation coefficients compared with the same percentages of the main matrix and filler bulk Bi_2_O_3_, at the same photon energies. Furthermore, the greater the amount of gypsum, the greater the mass attenuation coefficient for all weight percentages of Bi_2_O_3_ at different γ-ray photon energies, ranging from 0.0595 MeV to 1.3325 MeV. The HVL, TVL, MFP, and Z_eff_ values confirm the results that were obtained from the mass attenuation coefficient. The EABF and EBF, for all investigated composites, increase at low photon energies until they reach maximum values at intermediate photon energies, then, they decline at high photon energies. Moreover, the larger amounts of bismuth oxide composite show lower absorptions and exposure buildup factors. Finally, the greater the penetration depth, the higher the EABF and EBF values.

## Figures and Tables

**Table 1:** Chemical composition of bentonite and gypsum.

Metal Oxide	Weight Fraction (%)	
	Bentonite	Gypsum
Na_2_O	1.30	0
MgO	1.18	0
Al_2_O_3_	20.35	0
SiO_2_	49.65	0
SO_3_	1.96	41.27
K_2_O	1.28	0
CaO	10.92	58.73
TiO_2_	2.74	0
FeO	10.62	0

**Table 2:** The sample codes and the weight fraction in percentage (wt%) of bentonite, gypsum, and Bi_2_O_3_.

Sample Codes	Compositions (wt %)		
	Main Matrix		Bismuth Oxide (Bi_2_O_3_)
	Bentonite	Gypsum	
BG-0	67	33	0
BG-1		27	6
BG-2		20	13
BG-3	61	33	6
BG-4	54		13
BG-5	56.7	23.3	20

**Table 3:** Measured values and theoretical values using the XCOM program of mass attenuation coefficients, linear attenuation coefficients, relative deviation, and density of pure bentonite–gypsum, micro-Bi_2_O_3_/bentonite–gypsum, and nano-Bi_2_O_3_/bentonite–gypsum composites.

Sample Code/wt%	Energy (keV)	Mass Attenuation Coefficient (cm^2^ g^−1^)					Linear Attenuation Coefficient (cm^−1^)		Density (g cm^−3^)	
		(MAC)_XCOM_	(MAC)_Micro_	∆_1_ %	(MAC)_Nano_	∆_2_ %	(LAC)_Micro_	(LAC)_Nano_	Micro	Nano
BG-0 0 wt %	59.53	0.3580	0.3522	1.647			0.6861		1.948 ± 0.01	
	80.99	0.2238	0.2215	1.038			0.4315			
	356.01	0.0993	0.0979	1.430			0.1907			
	661.66	0.0766	0.0787	−2.668			0.1533			
	1173.23	0.0583	0.0586	−0.512			0.1142			
	1332.5	0.0547	0.0538	1.673			0.1048			
BG-1 6 wt %	59.53	0.6018	0.5858	2.731	0.6772	15.603	1.3473	1.5901	2.300 ± 0.02	2.348 ± 0.01
	80.99	0.3256	0.3277	−0.641	0.3631	10.803	0.7537	0.8526		
	356.01	0.1087	0.1104	−1.540	0.1183	7.156	0.2539	0.2778		
	661.66	0.0781	0.0759	2.899	0.0826	8.827	0.1746	0.1939		
	1173.23	0.0584	0.0571	2.277	0.0607	6.305	0.1313	0.1425		
	1332.5	0.0547	0.0531	3.013	0.0556	4.708	0.1221	0.1305		
BG-2 13 wt %	59.53	0.8863	0.8646	2.510	1.0051	16.250	2.1139	2.5831	2.445 ± 0.03	2.570 ± 0.02
	80.99	0.4442	0.4329	2.610	0.4932	13.929	1.0584	1.2675		
	356.01	0.1196	0.1170	2.222	0.1301	11.197	0.2861	0.3344		
	661.66	0.0799	0.0769	3.901	0.0844	9.753	0.1880	0.2169		
	1173.23	0.0585	0.0572	2.273	0.0613	7.168	0.1399	0.1575		
	1332.5	0.0547	0.0531	3.013	0.0556	4.708	0.1298	0.1429		
BG-3 6 wt %	59.53	0.6049	0.6008	0.682	0.6954	15.746	1.4209	1.6655	2.365 ± 0.02	2.395 ± 0.02
	80.99	0.3267	0.3209	1.807	0.3704	15.425	0.7589	0.8871		
	356.01	0.1088	0.1068	1.873	0.1304	22.097	0.2526	0.3123		
	661.66	0.0782	0.0783	−0.128	0.0851	8.685	0.1852	0.2038		
	1173.23	0.0584	0.0595	−1.849	0.0636	6.891	0.1407	0.1523		
	1332.5	0.0547	0.0544	0.551	0.0570	4.779	0.1287	0.1365		
BG-4 13 wt %	59.53	0.8930	0.9128	−2.169	1.0609	16.225	2.3094	2.8294	2.530 ± 0.02	2.667 ± 0.03
	80.99	0.4467	0.4512	−0.997	0.5137	13.852	1.1415	1.3700		
	356.01	0.1198	0.1213	−1.237	0.1348	11.129	0.3069	0.3595		
	661.66	0.0800	0.0816	−1.961	0.0895	9.681	0.2064	0.2387		
	1173.23	0.0586	0.0572	2.448	0.0616	7.692	0.1447	0.1643		
	1332.5	0.0548	0.0558	−1.792	0.0585	4.839	0.1412	0.1560		
BG-5 20 wt %	59.53	1.1760	1.1632	1.100	1.3286	14.219	3.2744	3.9300	2.815 ± 0.03	2.958 ± 0.01
	80.99	0.5649	0.5483	3.028	0.6204	13.150	1.5435	1.8351		
	356.01	0.1306	0.1324	−1.360	0.1492	12.689	0.3727	0.4413		
	661.66	0.0818	0.0831	−1.564	0.0923	11.071	0.2339	0.2730		
	1173.23	0.0587	0.0581	1.033	0.0639	9.983	0.1636	0.1890		
	1332.5	0.0547	0.0550	−0.545	0.0585	6.364	0.1548	0.1730		

**Table 4:** The effective atomic number (Z_eff_) of all tested specimens.

Energy (keV)	BG-0	BG-1	BG-2	BG-3	BG-4	BG-5
59.53	17.1943	49.5183	62.7007	49.6420	62.9440	69.1968
80.99	15.6597	42.7528	56.7242	42.7966	56.8427	64.3237
356.01	13.3757	24.6872	34.9620	24.7671	35.1137	43.1547
661.66	13.3390	19.4663	26.0590	19.5548	26.2427	32.2615
1173.23	13.3323	18.0070	23.2816	18.0978	23.4748	28.5231
1332.50	13.3346	17.8930	23.0561	17.9839	23.2498	28.2105

## Data Availability

All data are available in the manuscript.
